# Supplementary material for: Preparation of Poly(vinyl Alcohol) Microparticles for Freeze Protection of Sensitive Fruit Crops
Source: Polymers (Basel). 2022 Jun 16;14(12):2452. doi: 10.3390/polym14122452 (PMC9228911; doi:10.3390/polym14122452)
Supplement: Supplementary file 1 [file polymers-14-02452-s001.zip › polymers-1764875-supplementary.pdf]

Supplementary information

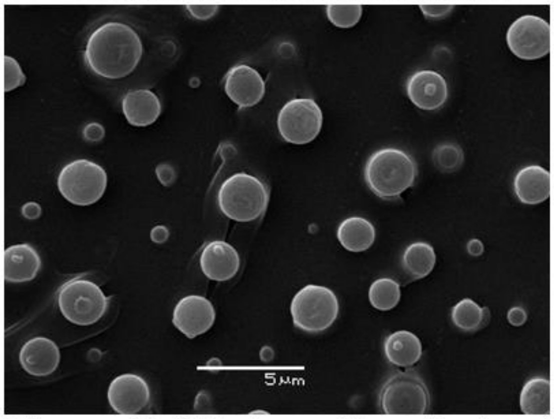

Figure S1-A. SEM image of L-PVA microparticles without neutralization

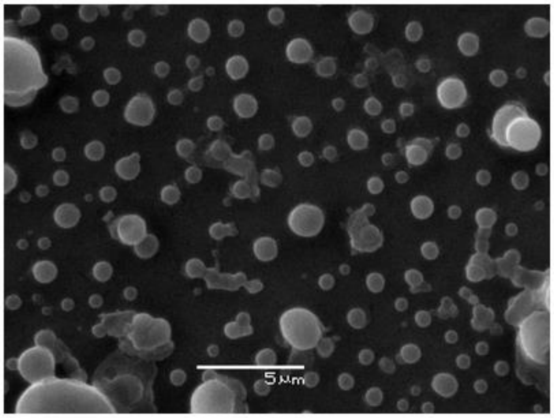

Figure S1-B. SEM image of m-PVA microparticles without neutralization

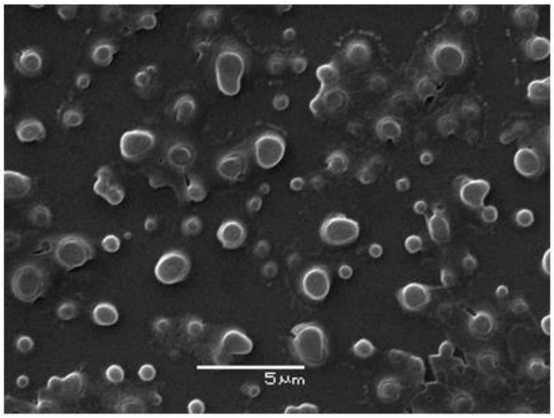

Figure S1-C. SEM image of h-PVA microparticles without neutralization

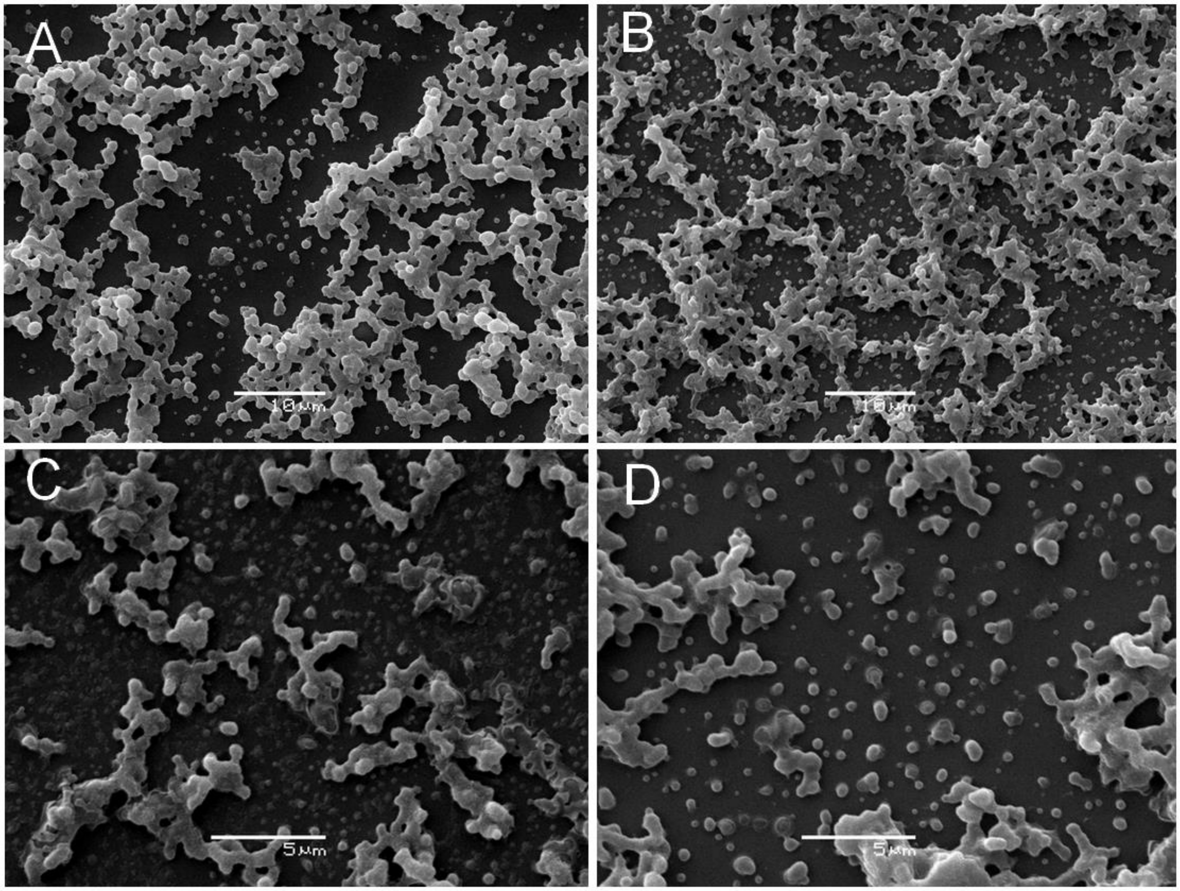

Figure S2. SEM images of h-PVA microparticles neutralized in different time storage. (A) and (C) 7 days of storage, (B) and (D) 180 days of storage.
